# Selective Single-Molecule Nanopore Detection of mpox A29 Protein Directly in Biofluids

**DOI:** 10.1021/acs.nanolett.3c02709

**Published:** 2023-12-05

**Authors:** Shenglin Cai, Ren Ren, Jiaxuan He, Xiaoyi Wang, Zheng Zhang, Zhaofeng Luo, Weihong Tan, Yuri Korchev, Joshua B. Edel, Aleksandar P. Ivanov

**Affiliations:** † Department of Chemistry, Imperial College London, Molecular Science Research Hub, White City Campus, 82 Wood Lane, London W12 0BZ, U.K.; ‡ Department of Metabolism, Digestion and Reproduction, Imperial College London, Hammersmith Campus, Du Cane Road, London W12 0NN, U.K.; § Nano Life Science Institute (WPI-NanoLSI), Kanazawa University, Kakuma-machi, Kanazawa 920-1192, Japan; ∥ The Key Laboratory of Zhejiang Province for Aptamers and Theranostics, Aptamer Selection Center, Hangzhou Institute of Medicine (HIM), Chinese Academy of Sciences, Hangzhou, Zhejiang 310022, People’s Republic of China

**Keywords:** single-molecule sensing, nanopore, molecular carrier, sandwich binding assay

## Abstract

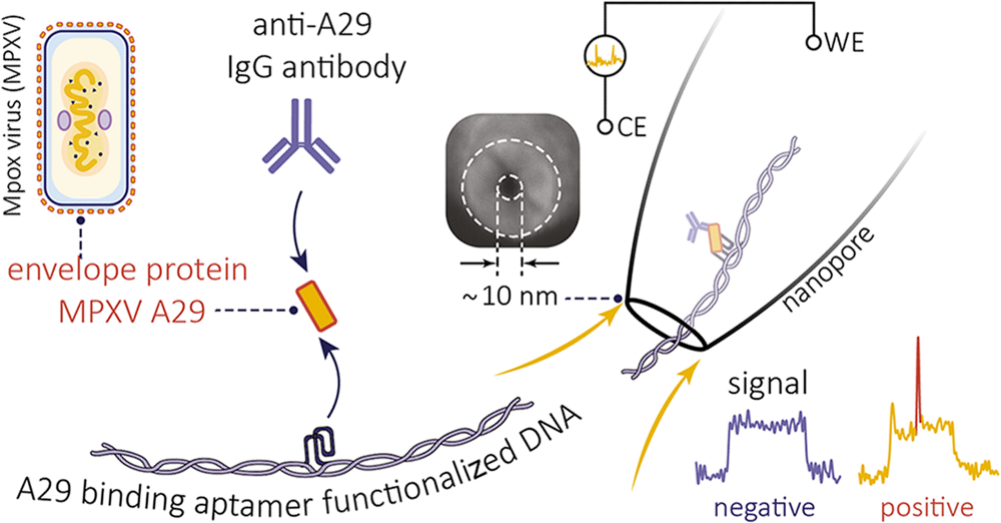

Single-molecule antigen detection using nanopores offers a promising alternative for accurate virus testing to contain their transmission. However, the selective and efficient identification of small viral proteins directly in human biofluids remains a challenge. Here, we report a nanopore sensing strategy based on a customized DNA molecular probe that combines an aptamer and an antibody to enhance the single-molecule detection of mpox virus (MPXV) A29 protein, a small protein with an M.W. of ca. 14 kDa. The formation of the aptamer–target–antibody sandwich structures enables efficient identification of targets when translocating through the nanopore. This technique can accurately detect A29 protein with a limit of detection of ∼11 fM and can distinguish the MPXV A29 from vaccinia virus A27 protein (a difference of only four amino acids) and Varicella Zoster Virus (VZV) protein directly in biofluids. The simplicity, high selectivity, and sensitivity of this approach have the potential to contribute to the diagnosis of viruses in point-of-care settings.

Mpox is a zoonotic viral disease caused by an *Orthopoxvirus*, called mpox virus (MPXV),^1^ which has started transmitting across multiple countries since May 2022 with 88026 cases being identified globally as of July 2023,^2^ and the outbreak was declared a Public Health Emergency of International Concern (PHEIC) by the world health organization (WHO) on 23 July 2022.^3^ Due to the relatively high case fatality ratio (around 3–6%) and transmissibility of mpox between humans,^4^ there is an urgency to develop rapid and accurate detection approaches that can be implemented for the containment of the spread.

A recent lesson learnt from the Covid-19 pandemic is that the development of easily accessible and cost-effective antigen detection can play a crucial role in preventing transmission of the virus.^5^ Unfortunately, since MPXV is serologically cross-reactive as multiple *Orthopoxviruses*,^4,6^ it is challenging to specifically distinguish MPXV from other pox viruses simply by using antigen/antibody detection. Although a few protein assay methods, such as enzyme-linked immunosorbent assays (ELISAs)^7^ and lateral flow assays (LFAs),^8^ are currently available, they have generally been limited by insufficient sensitivity at low viral loads.^7,9^ Currently, the accurate detection of MPXV relies on using a quantitative polymerase chain reaction (qPCR) to identify viral nucleic acids.^4,10,11^ However, qPCR testing is not commonly available in situ, which translates into significant processing time between sample collection and obtaining the test result, logistical challenges due to sample transportation, and increased testing costs.

Recent advancements in single-molecule detection have opened up new possibilities for highly sensitive and accurate detection of biomarkers.^12−14^ Among these advancements, nanopore sensing methods have garnered considerable attention due to their impressive capabilities in nucleic acid sequencing, molecular sensing, chemical catalysis, and characterization of individual molecules.^15−19^ In nanopore detection, analytes in electrolytic solution are translocated through a nanoscale pore under an applied electric field and characterized one at a time by recording the change in ion current passing through the pore during their translocation.^16,18,20−22^

However, since the changes in ion current are largely dependent on the volume and charge of the translocated analytes, selectivity is a common issue in distinguishing targets that are similar in size and surface charge. Various efforts have been made to address this issue, such as functionalizing the nanopore lumen with binding moieties^23−26^ or introducing receptor-bound molecular carriers made from DNA^27−31^ or nanomaterials.^32−36^ The integration of receptor-engineered DNA carriers has shown promise in facilitating the transport of protein biomarkers through the nanopore, enabling the specific detection of target proteins.^27,29^ However, this approach tends to be more effective for larger proteins where the secondary signal, superimposed on the DNA backbone, can be reliably detected. For smaller proteins, the signal-to-noise ratio may be insufficient, limiting the effectiveness of this approach.

Furthermore, in many nanopore sensing methods, detecting targets directly in unprocessed biological fluids poses a significant challenge due to the complexity and interference caused by the matrix. Despite progress made by some pilot studies,^37−39^ the requirement for time-consuming sample processing, extraction, and purification remains a common hurdle, limiting the potential applications of nanopore sensing for point-of-care (POC) use.

Here, we report a sensing strategy based on a DNA molecular probe that combines an aptamer and an antibody for single-molecule nanopore detection of small proteins directly in biofluids (Figure 1). We focus on the detection of MPXV A29 protein, a highly conserved surface envelope protein produced by the A29 gene of mpox virus, as the target. Traditionally, the relatively small size (14.4 kDa, 1.5–2 nm)^40^ of MPXV A29 protein and the high homology to the *Vaccinia* virus Copenhagen VACV A27 (a difference of only four amino acids)^41^ has posed additional challenges for its selective detection. In our strategy, an aptamer grafted onto the molecular probe specifically binds to the MPXV A29 protein. The aptamers used for targeting the MPXV A29 proteins were selected by using systematic evolution of ligands by exponential enrichment (SELEX). We selected the two highest affinity candidates, HIM-A29-5 and HIM-A29-6. An antibody was then bound to the MPXV A29 protein epitope, forming an aptamer–target–antibody sandwich structure (Figure 1a). When translocating through a nanopore, this structure is identifiable by its unique nanopore current signature, as illustrated in Figure 1. Importantly, this strategy is independent of the size of the target analyte, and we were able to quantify the MPXV A29 protein concentration with a dynamic range from 100 fM to 10 nM and a limit of detection (LOD) of as low as 11 fM. We show that the specificity is also improved, and we can distinguish MPXV A29 from VACV A27 protein (*Vaccinia* virus) and VZV protein (*Varicella zoster* virus) directly in biofluids.

**Figure 1:**
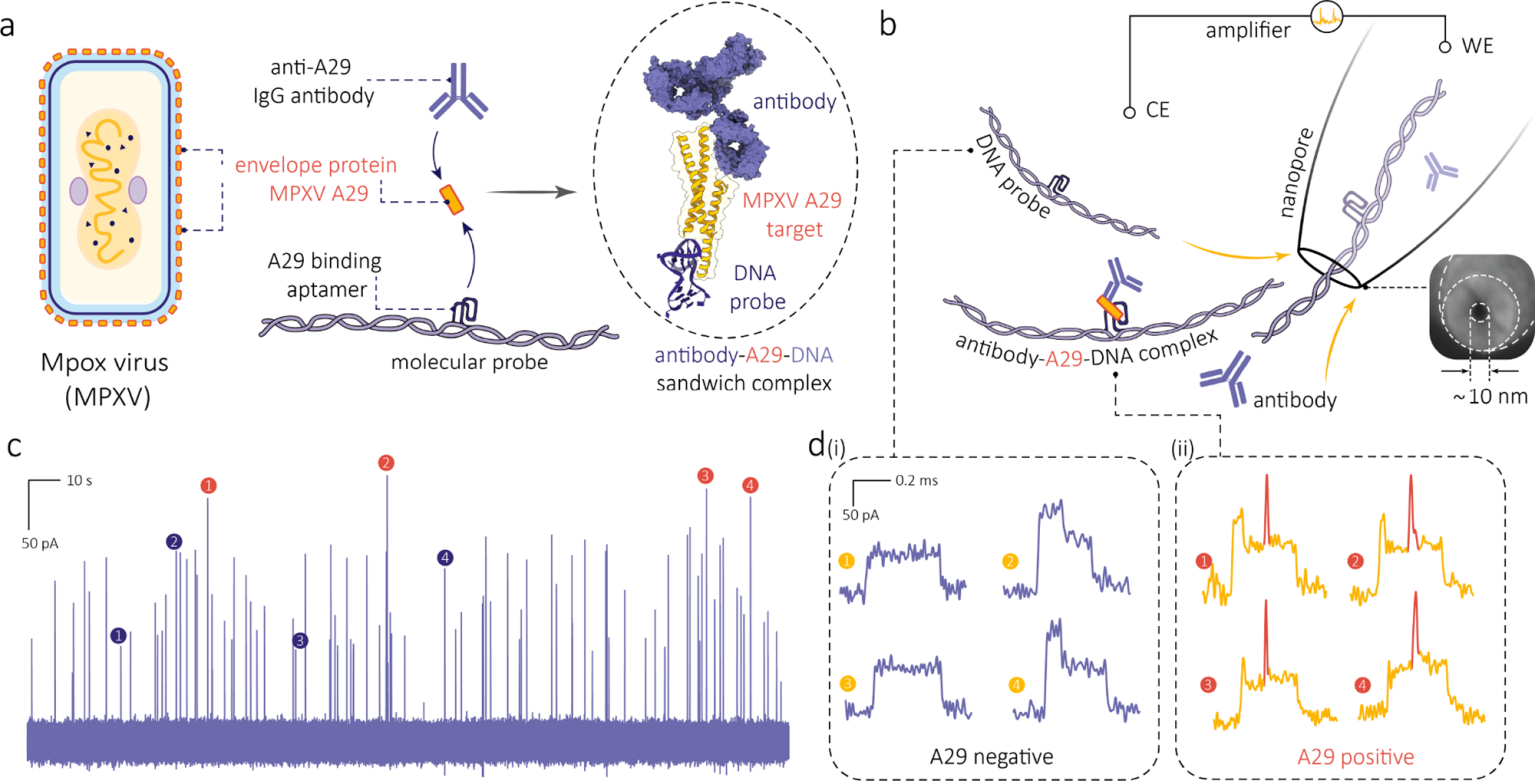
Single-molecule sensing of MPXV A29 protein through the utilization of an aptamer–target–antibody sandwich structure. (a) Schematic of envelope protein MPXV A29 forming a sandwich structure with the aptamer grafted on a DNA molecular probe and the antibody. (b, c) Schematic and representative current–time trace for the translocation of the aptamer-labeled molecular probe (100 pM) in the presence of A29 protein (100 pM) and its antibody (20 nM) through a nanopore. The inset in (b) shows an SEM image of a typical nanopore used in this work with an estimated size of 10 nm. (d) Typical events observed during the translocation experiments are shown, indicating the translocation of the molecular probe alone (i) and in the presence of the A29 protein (ii). The presence of the A29 protein results in the formation of an aptamer–A29–antibody sandwich structure, leading to a distinct secondary peak observed at the middle of each translocation event (colored in red). The translocation experiment was performed in 1 M LiCl and 1 M KCl electrolyte (5 mM MgCl_2_, 10 mM Tris-HCl, 1 mM EDTA, pH = 8) at an applied potential bias of 300 mV.

## Results and Discussion

### Molecular Probe Design and Aptamer Selection

**Figure 2:**
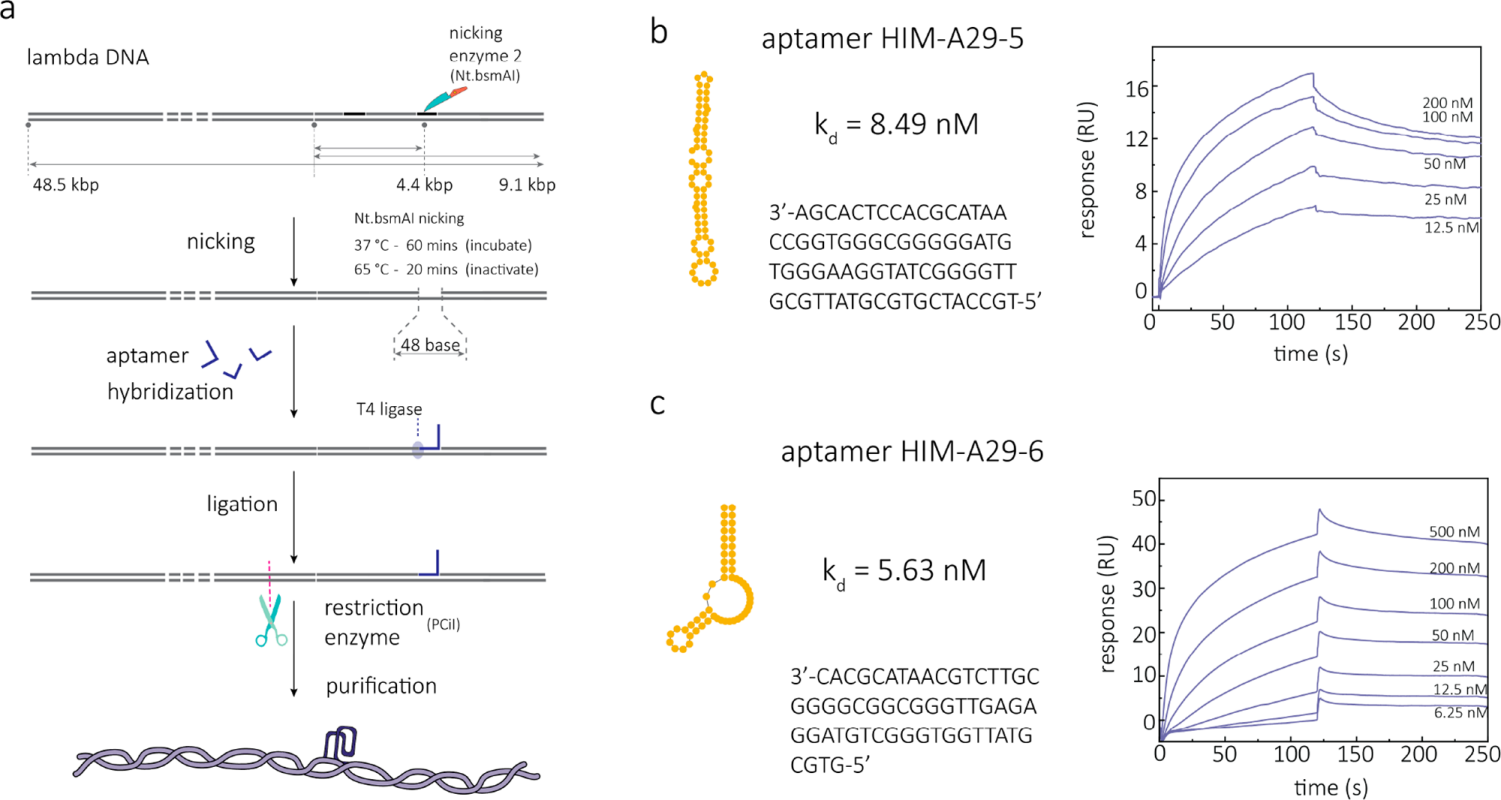
Molecular probe design and aptamer binding affinity. (a) Schematic illustration of the preparation process of the aptamer-grafted molecular probe (9.1 kbp) from a lambda-DNA. (b, c) Sequences and predicted structures of two aptamers (HIM-A29-5 and HIM-A29-6) used in this work and the surface plasmon resonance (SPR) response curves in the presence of different concentrations of A29 protein.

DNA molecular probes were made by using a 9.1 kbp dsDNA carrier. The aptamer sequence was grafted on the middle of the probe to minimize false positives due to DNA folding, which typically occurs at the ends of the probe. The 9.1 kbp molecular probe was made from lambda-DNA (48.5 kbp) using nicking and resctriction enzymes (Figure 2a and Figure S1). The nicking of DNA creates a 48-base single-stranded region, onto which the aptamer sequence was attached; see Methods in the Supporting Information.

Aptamers are single-stranded DNA/RNA oligonucleotides that can noncovalently bind their target molecules with high affinity and selectivity in the way the antibody does.^42^ DNA aptamer was chosen as the recognition probe in this study due to the advantages of small size, good stability, high tolerance of ambient environments, and ease of synthesis in vitro. The nature of the DNA sequence allows the aptamers to be easily tailored with any extended sequences at will and engineered into DNA nanocarriers as needed through a simple base-pairing hybridization binding chemistry.^43^ The aptamers used for targeting the MPXV A29 protein were selected by SELEX.^42,44^ We used the MPXV A29 protein as the target and exposed it to a constructed 76-mer ssDNA library that contains 36-nucleotide-long random sequences. Sequences were screened by SELEX to discover suitable aptamers that can specifically bind the target. An enriched pool containing sequences with increased affinity for the MPXV A29 protein was observed after seven rounds. qPCR measurments were performed to monitor the selection process. High-throughput sequencing methods were then used to identify the sequences of the enriched aptamer species in the pool (see Table S1 for the sequences). SPR measurements were used to determine the binding affinity between these aptamer candidates and MPXV A29 protein. We identified six aptamer candidates with high binding affinity (*k*_d_ smaller than 50 nM) (Figure 2b,c, Figure S2, and Table S2). Two candidates, named HIM-A29-5 and HIM-A29-6, with the highest binding affinity (*k*_d_ < 10 nM), were selected as the aptamers used in the following experiments (Figure 2b,c).

### Aptamer–Target–Antibody Sandwich Enhances the Identification of A29 by Nanopores

Nanopores were fabricated by laser-assisted pulling of quartz capillaries.^14,43,45^ The pulling protocol was optimized to generate nanopores with an average size of 9 ± 2 nm, as confirmed by scanning electron microscopy (SEM) images (Figure S3). These dimensions were in agreement with the values estimated through conductance measurements (18.67 ± 1.82 nS) conducted in a solution of 1 M LiCl and 1 M KCl (*n* = 20) (Figure S3).

An aqueous buffer solution of 1 M LiCl and 1 M KCl (with 5 mM MgCl_2_, 10 mM Tris-HCl, and 1 mM EDTA, pH = 8) was added to the bath, where a ground/reference Ag/AgCl electrode was placed. The same buffer solution was added inside the nanopipette, where an Ag/AgCl electrode was placed and set as the patch electrode (Figure 1b). The analytes were added to the bath (cis) and translocated through the nanopore inside the nanopipette (trans) by applying a positive clamped voltage. Chronoamperometric traces (*I*–*t*) were recorded by using a high-bandwidth amplifier sampled at 1 MHz and filtered by using a digital Bessel filter at 100 kHz.

**Figure 3:**
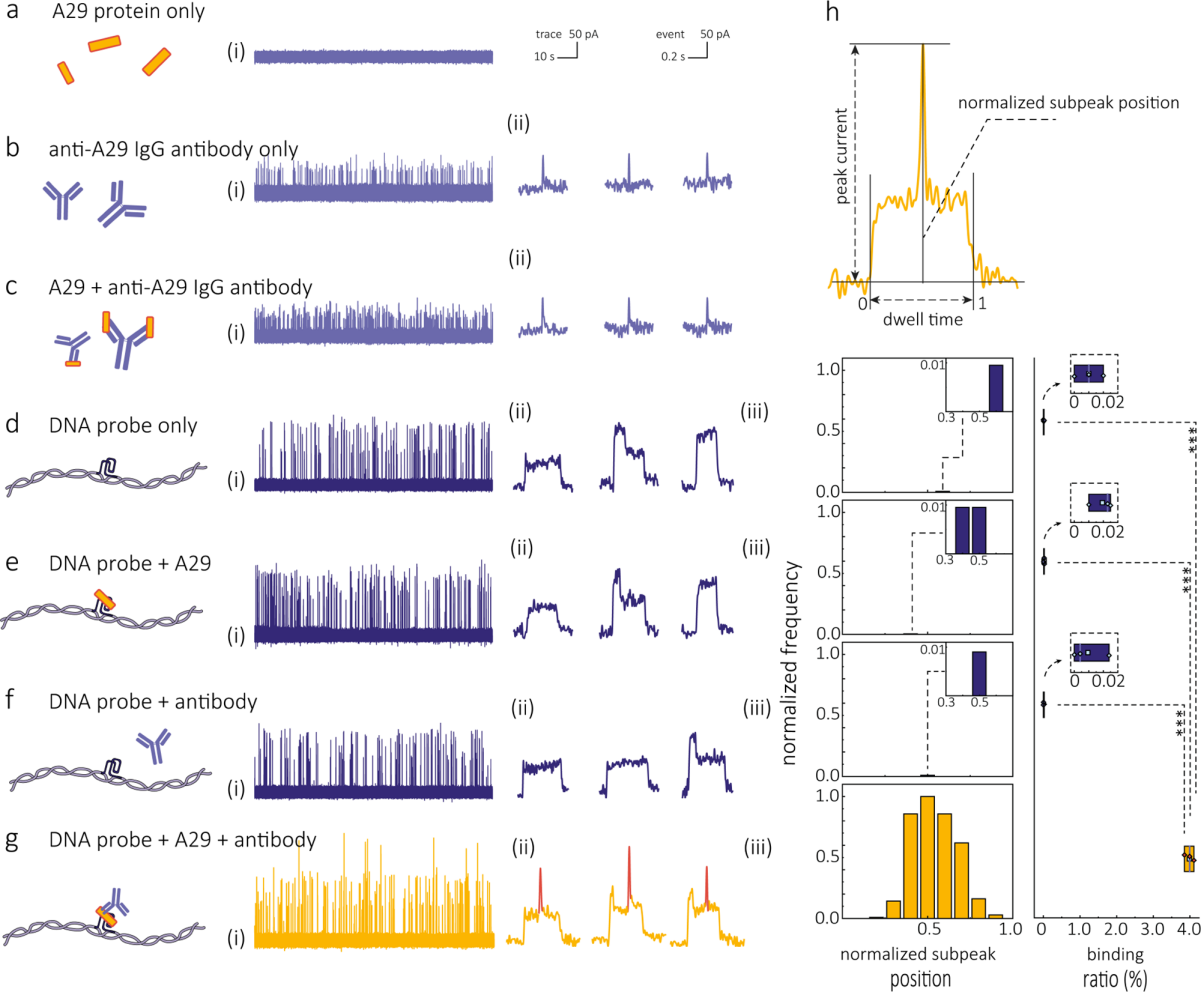
Aptamer–target–antibody sandwich structure and DNA molecular probe enhance the detection of A29 protein. (a-g) Schematics and representative current–time traces (i), typical events (ii), and statistics of normalized subpeak position and binding ratio (iii) for the translocation of (a) A29 protein only, (b) anti-A29 IgG antibody only, (c) A29 bound anti-A29 IgG antibody, (d) DNA molecular probe only, (e) DNA molecular probe + A29 protein, (f) DNA molecular probe + antibody, and (g) DNA molecular probe + A29 + antibody. The DNA probe, A29 protein, and antibody concentration were 100 pM, 1 nM, and 20 nM, respectively. (h) A representative bound event is shown, depicting the parameters of peak current, dwell time, and fractional position of the bound complex for the translocation of the DNA molecular probe with the aptamer–A29–antibody complex. All the translocation experiments were performed in 1 M LiCl and 1 M KCl electrolyte (5 mM MgCl_2_, 10 mM Tris-HCl, 1 mM EDTA, pH = 8) at an applied potential bias of 300 mV. Statistical significance was tested using a two-tailed Student’s *t* test. *p < 0.05; **p < 0.01; ***p < 0.001.

Control experiments were conducted independently with individual A29 protein, anti-A29 IgG, and A29 bound to anti-A29 IgG, without DNA molecular probes (Figure 3a–c). No translocation events were observed for the A29 protein alone due to its small size and fast translocation through nanopores (Figure 3a and Figure S4). On the other hand, translocation events were observed for the anti-A29 IgG (Figure 3b). These events, however, were indistinguishable from the A29 bound to anti-A29 IgG events (Figure 3c) as both type of events had similar dwell times (0.016 ± 0.005 ms vs 0.019 ± 0.004 ms) and peak amplitudes (0.087 ± 0.028 nA vs 0.075 ± 0.037 nA), This similarity can be linked to their comparable sizes, approximately 150 and 164 kDa, respectively (Figure S5a,b).

Subsequent control tests utilized the DNA molecular probe (100 pM) without the target protein MPXV A29. A typical signal shape, characteristic of the translocation of individual long DNA molecules through the nanopore, was observed (Figure 3d). The dwell time distribution averaged 0.42 ± 0.12 ms, and the peak current amplitude distribution had peaks of 0.132 ± 0.028 and 0.204 ± 0.054 nA, due to partial folding of the DNA molecular probe (66.76 ± 0.33%) (Figure S5c and Figure S6). When the MPXV A29 protein (1 nM) was introduced to the aptamer-modified molecular probe, translocation patterns mirrored those of the sole molecular probe. No discernible changes in the translocation signal shape (Figure 3(ii)) were observed during their translocation with a similar dwell time (0.42 ± 0.12 ms) and peak amplitude (0.123 ± 0.023 and 0.192 ± 0.046 nA). This lack of change was attributed to the small size of the A29 protein.

To enable the detection of MPXV A29 protein using nanopores, we introduced an antibody (anti-A29 IgG, approximately 150 kDa^46^) capable of binding to the MPXV A29 protein epitope, in addition to the aptamer. The combination of the antibody and aptamer formed a sandwich structure involving the molecular probe, aptamer, target protein, and antibody. In order to validate the formation of the sandwich complex structure, we investigated the binding kinetics of the A29 protein with the aptamer and anti-A29 IgG. SPR analysis provided a sensorgram (Figure S7) that demonstrated the sequential capturing of the aptamer (HIM-A29-6) and anti-A29 IgG by the A29 protein. This sequential binding indicated that the aptamer and antibody interacted with distinct binding sites on the A29 protein. As a result, a ternary complex consisting of the aptamer, A29 protein, and antibody was formed, establishing the aptamer–A29–antibody sandwich structure. It should be noted that there was no SPR response when the sandwich structure formation was tested with the HIM-A29-5 aptamer. Therefore, we used the HIM-A29-6 sequence for the following experiments.

Nanopore experiments were performed with the molecular probe in the presence of A29 (100 pM) and anti-A29 IgG (20 nM), as shown in Figure 3g. The considerably larger size of the anti-A29 IgG compared to the MPXV A29 protein (150 kDa vs. 14.4 kDa) resulted to a distinct secondary ion current peak emerging in the center of the detected events. This secondary peak indicated the presence of the formed aptamer–A29–antibody complex. A custom MATLAB App was developed to identify these secondary peaks (see Data Analysis in the Supporting Information). The relative position of the bound protein along the DNA molecular probe was measured as a fraction of the normalized length of the DNA signal (Figure 3h). This normalization is necessary to eliminate the impact of dwell time variation arising from stochastic translocation for each molecular probe.^20,47^ To avoid counting false positives associated with folded events, we isolated positive events by setting the following thresholds (Figure S8): (1) fractional position between 0.5 ± 0.2, to take into account the sandwich structure bound in the middle position in an unfold DNA, (2) secondary peak width less than 0.1 ms, and (3) peak height larger than μ ± 4σ, where μ represents the mean value for the DNA unfolding level where σ represents the standard deviation (Figure S8). It should be noted that when the translocation event exhibited both folding and protein binding signals, these events were individually examined to verify that all binding events were classified correctly.

We observed a significant increase in subpeak binding ratio (3.98 ± 0.11%) for the translocation of molecular probes in the presence of A29 target and anti-A29 IgG (Figure 3g(iii)) This is in contrast to the ratios for the DNA molecular probes alone (0.01 ± 0.01%, Figure 3d(iii)) and DNA molecular probe with A29 protein (0.02 ± 0.01, Figure 3e(iii)). Control experiments involving the DNA molecular probe and the antibody alone, without the A29 protein, showed a negligible secondary peak ratio (0.01 ± 0.01%, Figure 3f(iii)). They also showed comparable translocation dwell time (0.42–0.44 ms) and peak amplitude (0.125–0.135 nA for unfolded DNA and 0.195–0.205 nA for folded DNA), (Figure S5c–e), confirming that the secondary peaks originated from the formation of the aptamer–A29–antibody complex. The binding of the molecular probe to the A29 protein and antibody complex did not significantly impact the overall dwell time (0.43 ± 0.13 ms, Figure S5); however, we observed a slightly higher amplitude (0.148 ± 0.098 nA) for these secondary peaks. Still, these binding events can be precisely identified through the aforementioned subpeak detection. These findings confirmed that using DNA molecular probes with the aptamer–target–antibody sandwich configuration facilitates the selective detection of small protein targets via nanopore analysis.

### Single-molecule Quantification of MPXV A29 Protein

**Figure 4:**
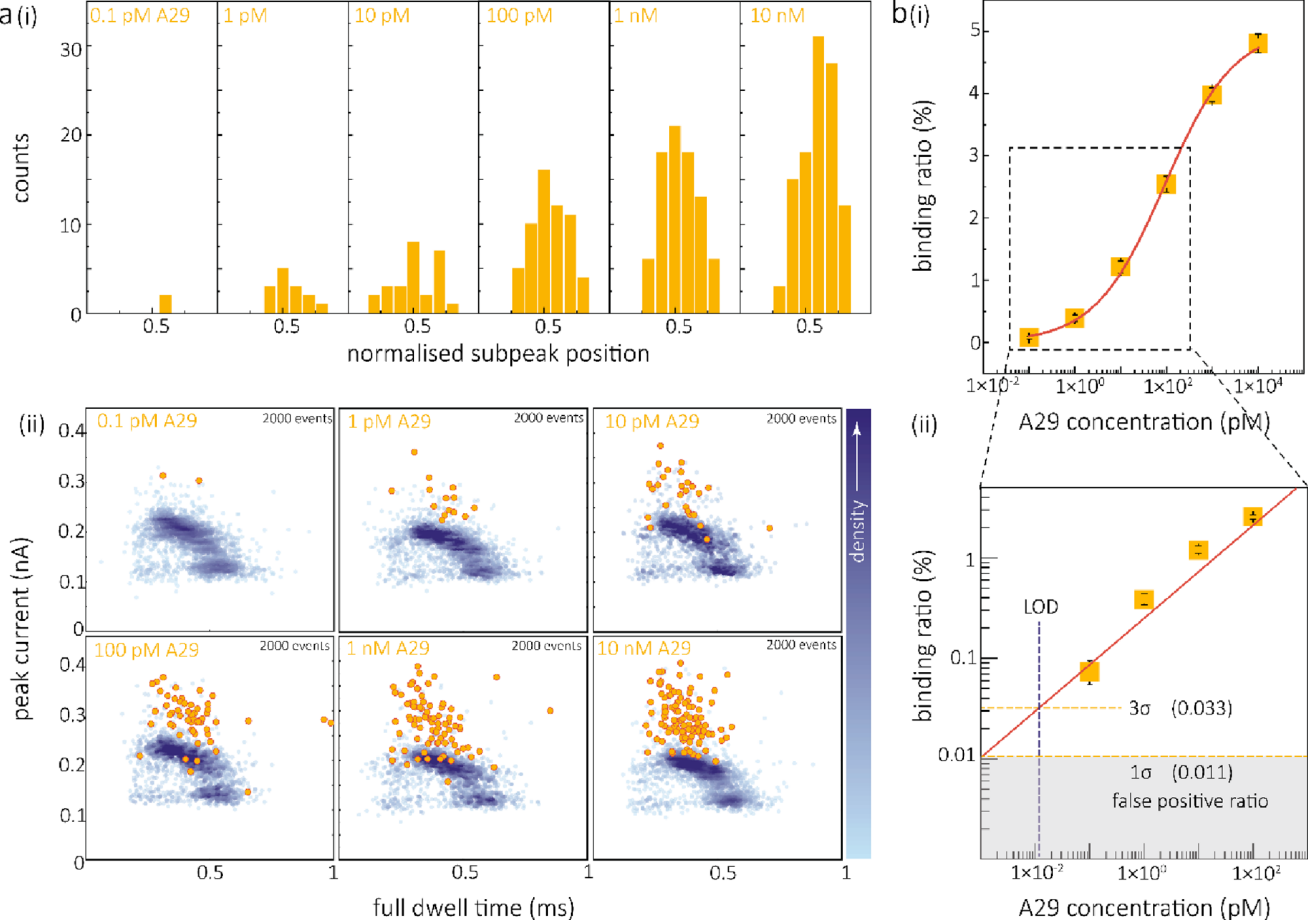
Single-molecule quantification of the A29 protein and the sensitivity of the detection method. (a) Statistics of normalized fractional position of peaks for A29 protein ranging from concentrations of 0.1 pM to 10 nM (i). The scatter plots of peak current and dwell time for the translocation events of the DNA molecular probe (100 pM) and antibody (20 nM) in the presence of A29 protein concentrations ranging from 0.1 pM to 10 nM are shown in (ii). Events with an A29 bound peak are highlighted in yellow. The total number of events recorded for each concentration is 2,000. (b) The binding curve (i) demonstrating the interaction of the molecular probe with A29 protein at concentrations ranging from 0.1 pM to 10 nM in the presence of a 20 nM antibody. The zoom-in view (ii) shows a linear fitting from 0.1 to 100 pM of A29 protein and the calculation of LOD-based 3σ of the blank control. The blank control experiments (*n* = 7) were performed with all the same solutions but without target A29 protein, namely, at the same concentrations of DNA molecular probe and antibody. All translocation experiments were conducted in an electrolyte containing 1 M LiCl, 1 M KCl, 5 mM MgCl_2_, 10 mM Tris-HCl, and 1 mM EDTA, at a pH of 8, with an applied bias potential of 300 mV. The error bars in (b) represent the standard deviation obtained from three independent replicates.

To investigate the concentration-dependent behavior of MPXV A29 protein, we conducted a study encompassing concentrations ranging from 0.1 pM to 10 nM. In each experiment, we recorded and statistically analyzed a minimum of 2000 translocation events. As expected, the number of events exhibiting a secondary peak situated in the middle region (highlighted in yellow) increased with higher concentrations of A29, as depicted in Figure 4a. Scatter plots illustrated that most of the bound events displayed a higher peak amplitude (0.27 ± 0.13 nA) compared to the unbound events (0.12 ± 0.03 nA for the unfolded signal and 0.19 ± 0.04 nA for the folded signal), as shown in Figure 4a(ii). However, by utilizing an algorithm that identifies the secondary peak in the corresponding position, we effectively avoid excluding positive results with peak heights identical to those of folded DNA, thereby enhancing the accuracy of detection (see Data Analysis in the Supporting Information).

The correlation between A29 binding and its concentration was established by plotting the ratio of binding events to the total number of translocation events for the DNA molecular probes (Figure 4b). By fitting the curve with the Hill function as reported previously,^43^ we obtained a binding affinity of *K*_d_ = 82.3 pM. This value was approximately 1 order of magnitude smaller than the *K*_d_ value obtained from bulk SPR measurements (Figure 2c). The smaller dissociation constant could be attributed to factors such as (i) analyte molecules might slow down when they arrive near the tip of the nanopipette^48^ and (ii) the significant reduction in DNA migration caused by electro-osmotic flow (EOF) at the pipette tip, resulting in the accumulation of analytes in the vicinity of the nanopore.^49,50^

The detection method exhibited high efficiency over a broad dynamic range spanning 5 orders of magnitude (100 fM to 10 nM, Figure 4b). Due to the protein binding signals originating from the ternary sandwich structure formed by the target, false positives were relatively infrequent in the absence of the A29 target protein. As a result, LOD as low as 11 fM was determined using the calibration curve based on the value of the signal above 3σ of the blank background control (experiments without target protein; see Figure 4b(ii)). This level of sensitivity is particularly advantageous for the detection of rare proteins, and it is noteworthy that no purification steps were necessary.

Although not investigated in this study, sensitivity can be further enhanced by increasing the capture rate^51−53^ or employing other strategies such as target molecule preconcentration^38,39,54^ or paralleling multiple nanopore detection.^55^

### Specificity of MPXV A29 Protein Sensing in Human Serum and Saliva

**Figure 5:**
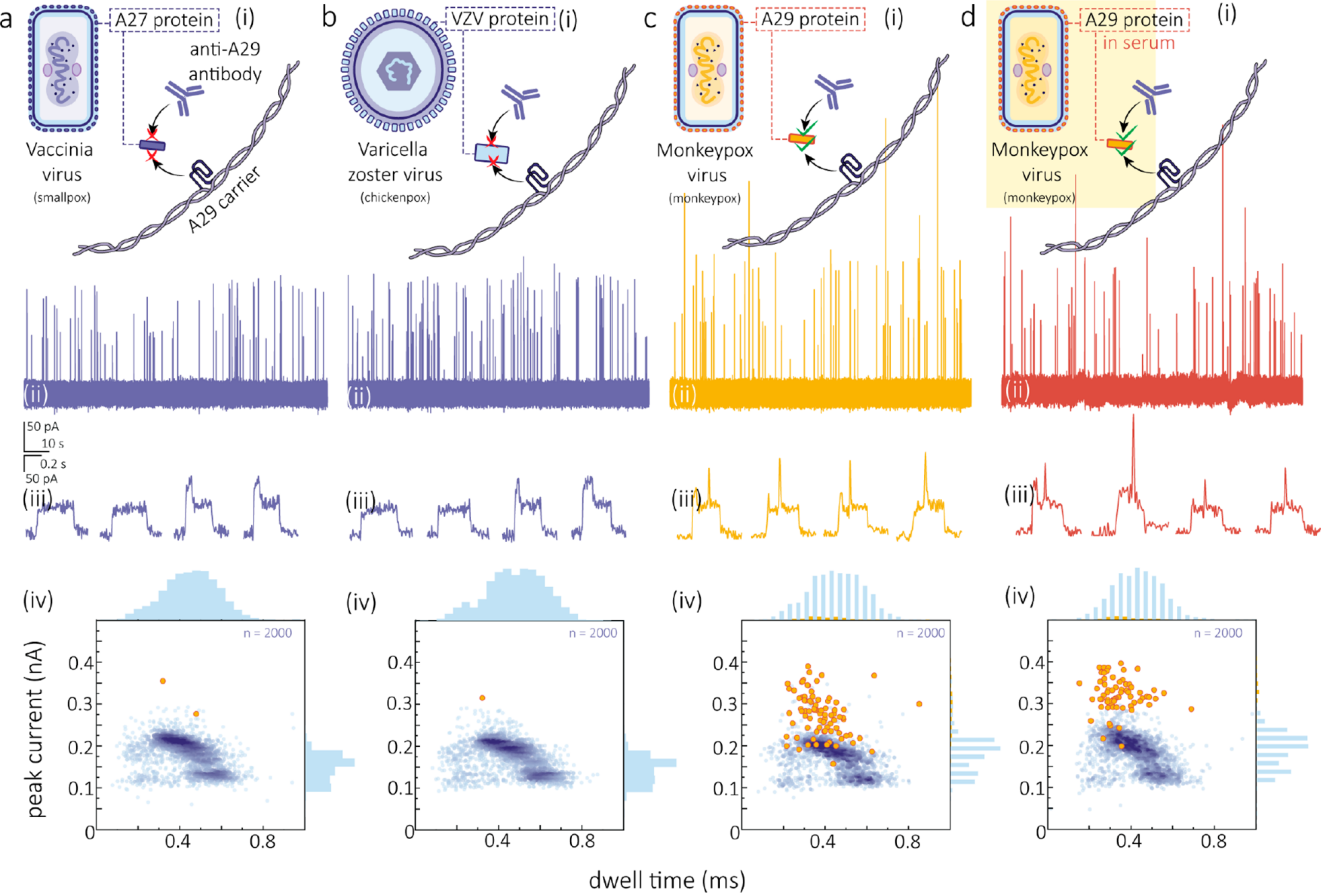
Specificity of MPXV A29 protein senising in human biofluids. (a-d) Schematics (i), representative current–time traces (ii), typical events (iii), and statistics of dwell time and peak current (iv) for the translocation of DNA molecular probes in the presence of *Vaccinia* virus A27 protein (a), *Varicella zoster* virus protein (b), MPXV A29 protein (c), and MPXV A29 protein within serum (d). The translocation events with detected peaks are highlighted in yellow (iv). The final concentrations of DNA probe, target protein, and antibody were 100 pM, 1 nM, and 20 nM, respectively, in all experiments. Measurements in (a–c) were performed in 1 M LiCl and 1 M KCl electrolyte (5 mM MgCl_2_, 10 mM Tris-HCl, 1 mM EDTA, pH = 8), while measurement in (d) was performed in the above electrolyte mixed with human serum at a 20:1 ratio. All the applied potential biases were 300 mV.

MPXV, as one of the *Orthopoxviruses* in the Poxviridae family, exhibits considerable antigenic protein homology with other species within the family. This similarity can potentially lead to false positives and misdiagnosis.^8,41^ To assess the specificity of our detection method, we conducted control experiments using the envelope protein A27 from *Vaccinia* virus (14.46 kDa), which shares significant homology with MPXV A29 protein, differing only in four amino acids.^41^ Additionally, we selected the VZV protein (78 kDa) from *Varicella zoster* virus (chickenpox) as another control, since the VZV is still widely transmitting around the world to date and can complicate the identification of MPXV. Nanopore sensing was performed by introducing DNA molecular probes (100 pM), an excess of anti-A29 antibody (20 nM), and one of the antigen proteins being tested: A27 protein, VZV protein, or A29 protein (1 nM) (Figure 5a–c). For the A27 and VZV proteins, almost no secondary peaks were observed in the translocation events, indicating a negligible binding ratio (0.01 ± 0.01% for A27 protein, 0.00 ± 0.01% for VZV protein) (Figure 5a,b and Figure S9). In contrast, a significant increase in the binding ratio was observed in the presence of A29 protein (3.98 ± 1.11%, with a *p* value <0.001 for A27 and <0.0001 for VZV protein). The high selectivity of the detection method can be attributed to the requirement for simultaneous binding of the target molecule to both the aptamer and the antibody, forming a sandwich-like structure. In contrast, nontarget molecules typically exhibit weaker binding to both the aptamer and the antibody, resulting in fewer detectable binding events.

For practical applications in clinical settings, it is essential that a biosensor can work directly in complex biological matrices.^14^ To evaluate the specificity of our biosensor toward MPXV A29 protein in unprocessed biological fluids, we conducted nanopore detection experiments using human serum and saliva, where genetic materials of MPXV have been previously detected.^56,57^ Serum and saliva samples were spiked with MPXV A29 protein and mixed with the translocation buffer at a ratio of 1:20, comprising 100 pM DNA molecular probe and 10 nM anti-A29 antibody, as described in Methods in the Supporting Information. Following incubation, nanopore experiments were directly performed under the same conditions as those in the buffer.

For the A27 protein (1 nM) and VZV protein (1 nM) in both serum and saliva, no translocation events with peaks in the middle were observed. The current–time traces and representative translocation signals are presented in Figures S10 and S12. The binding ratio was minimal, with 0.02 ± 0.01% and 0.01 ± 0.01% for A27 protein and 0.02 ± 0.02% and 0.00 ± 0.01% for VZV protein, in serum and saliva, respectively (Figures S10–S13). In contrast, a significant proportion of binding events was observed when the sensor was tested with MPXV A29 protein in both serum (3.53 ± 0.12%, with a *p* value <0.001 for A27 protein and <0.001 for VZV protein) and saliva (3.21 ± 0.13%, with a *p* value <0.001 for A27 protein and <0.0001 for VZV protein) (Figure 5d and Figures S10–S13). These results confirm that the MPXV A29 protein can be directly detected with high specificity in unprocessed human serum and saliva.

## Conclusions

We have successfully demonstrated a highly sensitive and specific nanopore-based single-molecule detection strategy using a customizable DNA molecular probe for quantifying the mpox virus A29 protein. Our sensing approach leverages the aptamer–target–antibody sandwich structure, which greatly enhances the detection of small protein bindings, such as the 14 kDa A29 protein from the mpox virus, through nanopore analysis. The developed nanopore sensor exhibits high sensitivity, achieving an LOD as low as 11 fM, without the need for target preconcentration or amplification. Additionally, we have demonstrated the capability of specifically detecting the A29 protein directly from unprocessed biological samples including human serum and saliva.

These findings suggest that accurate detection of highly transmittable viruses using nanopore technology can be rapidly deployed for future POC applications. However, it is important to acknowledge that further clinical validation and optimization are necessary before this strategy can be effectively employed for on-site diagnosis.

## Data Availability

The main data that support the results in this study are available within the paper and the Supporting Information or are available for research purposes from the corresponding authors upon reasonable request.

## Supporting Information Available

Preparation of aptamer-modified DNA molecular probes, separation, extraction, and purification of molecular probes, aptamer selection, SPR measurements, fabrication of nanopores, translocation experiments, and data acquisition (PDF)
