# Supplementary material for: Selective Single-Molecule Nanopore Detection of mpox A29 Protein Directly in Biofluids
Source: Nano Lett. 2023 Dec 5;23(24):11438–46. doi: 10.1021/acs.nanolett.3c02709 (PMC10755749; doi:10.1021/acs.nanolett.3c02709)
Supplement: Supplementary file 1 — nl3c02709_si_001.pdf [file nl3c02709_si_001.pdf]

Selective single-molecule nanopore detection of mpox A29 protein directly in biofluids

Shenglin Cai[1,#], Ren Ren[1,2,3,#,], Jiaxuan He[4,#], Xiaoyi Wang[1], Zheng Zhang[4], Zhaofeng, Luo[4], Weihong Tan[4,*], Yuri Korchev[2,3], Joshua B. Edel[1,*], Aleksandar P. Ivanov[1,*]*

[1]Department of Chemistry, Imperial College London, Molecular Science Research Hub, White City Campus, 82 Wood Lane, London W12 0BZ, UK.

[2]Department of Metabolism, Digestion and Reproduction, Imperial College London, Hammersmith Campus, Du Cane Road, London W12 0NN, UK.

[3]Nano Life Science Institute (WPI-NanoLSI), Kanazawa University, Kakuma-machi, Kanazawa 920-1192, Japan.

[4]The Key Laboratory of Zhejiang Province for Aptamers and Theranostics, Aptamer Selection Center, Hangzhou Institute of Medicine (HIM), Chinese Academy of Sciences, Hangzhou, Zhejiang 310022, China

*Correspondence: joshua.edel@imperial.ac.uk; alex.ivanov@imperial.ac.uk; r.ren15@imperial.ac.uk; tan@hnu.edu.cn

[#]These authors contributed equally: Shenglin Cai, Ren Ren, and Jiaxuan He

Methods

Preparation of aptamer-modified DNA molecular probes

The 9.1 kbp DNA molecular probe was prepared and edited from the lambda-phage DNA (λ-DNA, 48502 base pairs) using a set of commercially available enzymes through a multi-step reaction including nicking step, aptamer displacement, ligation, and digestion. The procedure is shown in Fig. 2a. For a typical reaction, the nicking was performed by incubating 12.5 µl of λ-DNA (stock, 15.8 nM) with 3.2 µl of Nb.BtsI (10 units/µl, NEB) in the presence of 5 µl of 10× rCutSmart buffer (NEB) and 29.3 µl of nuclease-free water (Thermo Fisher) at 37°C for 60 min, followed by inactivating at 80 °C for 20 min. The nicking process generates a 45-base breach at the position of ~4.7 kbp of the λ-DNA. The 48-base breach was then displaced by a designed aptamer probe, which contains a complementary sequence to the breach in the 5′ end (the sequence of the aptamer probe and the 48-base breach can be found in Table S3). The displacement was carried out by adding 1 µl of 20 µM aptamer probe (100× excess) through a customized hybridization protocol: heated to 75 °C for 5 min, gradually cooled down to 15 °C at a rate of 1 °C/min, and held at 4 °C. The resultant product was subsequently ligated by adding 3.6 µl of T4 DNA ligase (400 units/µl, NEB) and 6 µl of T4 ligation buffer (NEB), followed by incubating at 22 °C for 2 hours and inactivating at 65 °C for 15 min. Finally, 3.2 µl of PciI (units/µl, NEB) was added to digest the λ-DNA at the position of ~9.1 kbp.

Separation, extraction, and purification of molecular probes

The resultant 9.1 kbp DNA molecular probe was separated through a 0.7% (w%) agarose gel electrophoresis at a 4V/cm field strength for 120 min. As shown in Fig. S1a, the target band was then cut carefully and transferred into a 2 ml centrifuge tube. The target DNA was extracted and purified using a commercial gel extraction kit (T1020L, Monarch DNA Gel Extraction Kit, NEB) according to the supplier's protocol. The concentration of the purified molecular probe was determined by measuring the absorbance UV-Vis absorbance at 260 nm (Fig. S1b) using Nanodrop 2000c (Thermo SCIENTIFIC). The molecular probe was stored at -20 °C before use.

Aptamer selection

The A29 protein was coupled with NHS-activated carboxylic acid dynabeads (Invitrogen) following a reported procedure. Because the A29 protein has a His-tag, we used his-tag as the counter SELEX target. The efficiency of protein immobilization was quantified using flow cytometry with APC-labeled anti-his antibody and APC-labeled IgG antibody. The library was a 76nt length ssDNA with 36 randomized nucleotides and two 20-base primers. The library was denatured by heating at 95 °C for 10 minutes, then cooled on ice for 10 minutes. The His-coated beads and A29-coated beads were washed three times with 200 µL of DPBS buffer (with 5 mM Mg2+) and incubated with denatured library separately in DPBS (with 5 mM Mg2+, 0.1-1 mg/mL BSA, 0.1-1 mg/mL HS-DNA (Herring Sperm DNA), 0-0.02% tween-20) in room temperature. The A29-coated beads were washed with 200 µL of DPBS buffer (with 5 mM Mg^{2+}, 0.1-1 mg/mL BSA, 0.1-1 mg/mL HS-DNA, 0-2‱ tween-20, 0.1-0.2 mg/mL His) to remove unbound and weakly bound sequences. The A29-coated beads were collected in a final volume of 100 µL at the end of the selection. The eluted aptamers were amplified by PCR to generate ssDNA for next-round selection. The

selection was finished after 7 rounds. The candidate aptamers were characterized by high-throughput sequencing and SPR measurements.

SPR measurements

The surface plasmon resonance (SPR) measurements were conducted using a Biacore 8K system. The experimental setup involved immobilizing the A29 protein and His-tag on separate channels of the CM5 chip, which consisted of carboxymethylated dextran attached to a gold surface. Candidate sequences were individually tested at a concentration of 500 nM to identify high-affinity aptamers. The dissociation constant of the high-affinity aptamers was determined by performing measurements with different concentrations and subsequently fitting the data using the Evaluation Software. The data was fit to a simple Langmuir binding model using global kinetic rate constants for dissociation (k_d), association (k_a), and Rmax values per sample. The equilibrium dissociation (or affinity) constant (K_D) was calculated by determining the ratio of the kinetic rate constants, $K_D = k_d/k_a$.

Fabrication of nanopores

All nanopores used in this work were fabricated by pulling quartz capillaries (GQF100-50-7.5, World Precision Instruments, UK) through a laser-assisted pipette puller (Sutter Instrument, P-2000, USA). The pulling protocol used was slightly optimized according to previously reported[1-3]. In brief, capillaries (inner diameter: 0.5 mm, outer diameter: 1.0 mm, length: 7.5 cm) were thoroughly cleaned for 30 min using a plasma cleaner (Harrick Plasma) to remove any organic residues or contaminants. The pulling was then performed using a two-step parameter as follows: (1) HEAT: 775; FIL: 4; VEL: 30; DEL: 170; PUL: 80, (2) HEAT: 825; FIL: 3; VEL: 20; DEL: 145; PUL: 180. The humidity and temperature for the pulling condition were kept at ~30% and 21 °C to ensure the same pore geometry. It may need to re-optimize the protocol when the ambient conditions change. The average pore diameter fabricated in this work was 9 ± 2 nm (n = 5) based on the SEM measurement and conductance calculation, Fig. S3.

Translocation experiments

The nanopore translocation experiments were conducted in a high-salt concentration electrolyte solution containing 1 M LiCl, 1 M KCl, 5 mM MgCl2, 10 mM Tris-HCl, and 1 mM EDTA at pH 8. The DNA molecular probes, antibodies, and/or targets were added to an electrolyte bath (200 µl). A nanopipette containing only the electrolyte, with a volume of approximately 10 µl, was inserted in this bath. Two silver/silver chloride (Ag/AgCl) electrodes were placed in the nanopipette and bath, respectively, as the patch and bath/ground electrodes. The voltage bias was applied using a high-bandwidth amplifier (VC100, Chimera Instruments).

Prior to nanopore detection, the A29 protein (Catalog No. abx620117, Abbexa Ltd.) or control samples at specified concentrations were incubated with a 100 pM molecular probe and a 20 nM A29 antibody (Catalog No. 40891-M0036, Sino Biological, Inc.) for two hours in the electrolyte. The 100 pM concentration of the DNA molecular probe was used according to the optimization of previous work[4]. In experiments involving human serum (H4522, Sigma-Aldrich) and saliva (pooled from >3 healthy

volunteers and filtered with 2.2 µm filter (Thermo Fisher), the A27 (Cat: 40897-V07E, Sino Biological, Inc.), VZV (Catalog No. abx670269, Abbexa Ltd.), or A29 protein was spiked into the serum or saliva at predetermined final concentrations. The spiked samples were mixed with the molecular probes (final concentration of 100 pM) and A29 antibody (final concentration of 20 nM) in the electrolyte at a ratio of 1:20. Subsequently, the samples were incubated at room temperature for two hours before being transferred for nanopore detection.

<u>Data acquisition</u>

The current-time traces were recorded at a sampling rate of 1 MHz and then filtered at 100 kHz using a digital Bessel filter. Data analysis was carried out using a custom MATLAB code. The data analysis section of the Supporting Information provides a detailed description of the data analysis.

The current-time traces used in this study were directly exported from MATLAB, and plots and charts were created using Origin 2021b. All measurements were independently performed in triplicates, and the reported values represent the mean of the three measurements. The reported errors in this study correspond to one standard deviation obtained from three measurements.

Supplementary Figures

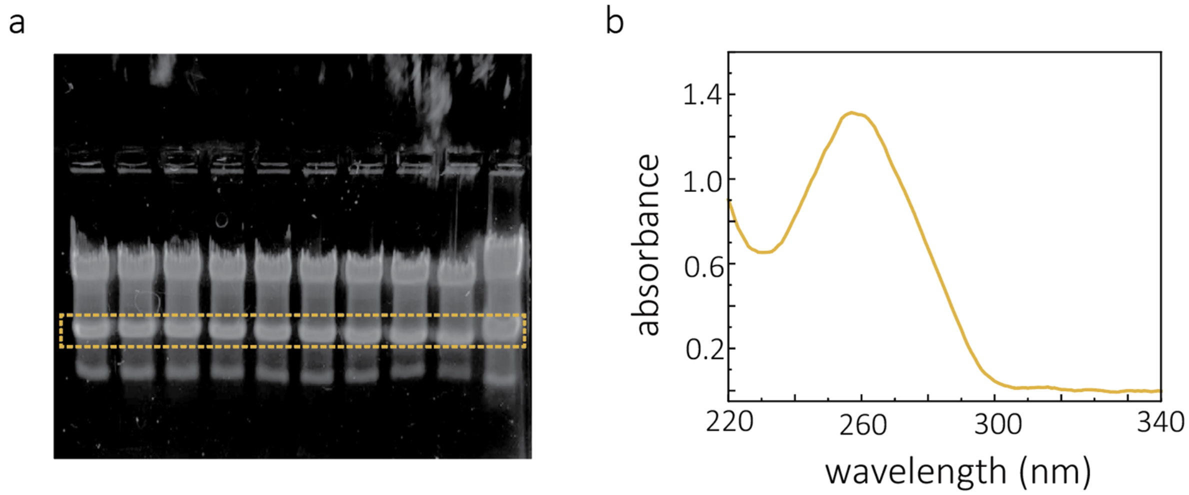

Fig. S1. (**a**) Gel electrophoresis showing the separation of the target DNA molecular probe (9.1 kbp) from other fragments. (**b**) UV-vis spectrum for the DNA molecular probe extracted from the gel. The concentration of the molecular probe was determined using the absorbance at 260 nm.

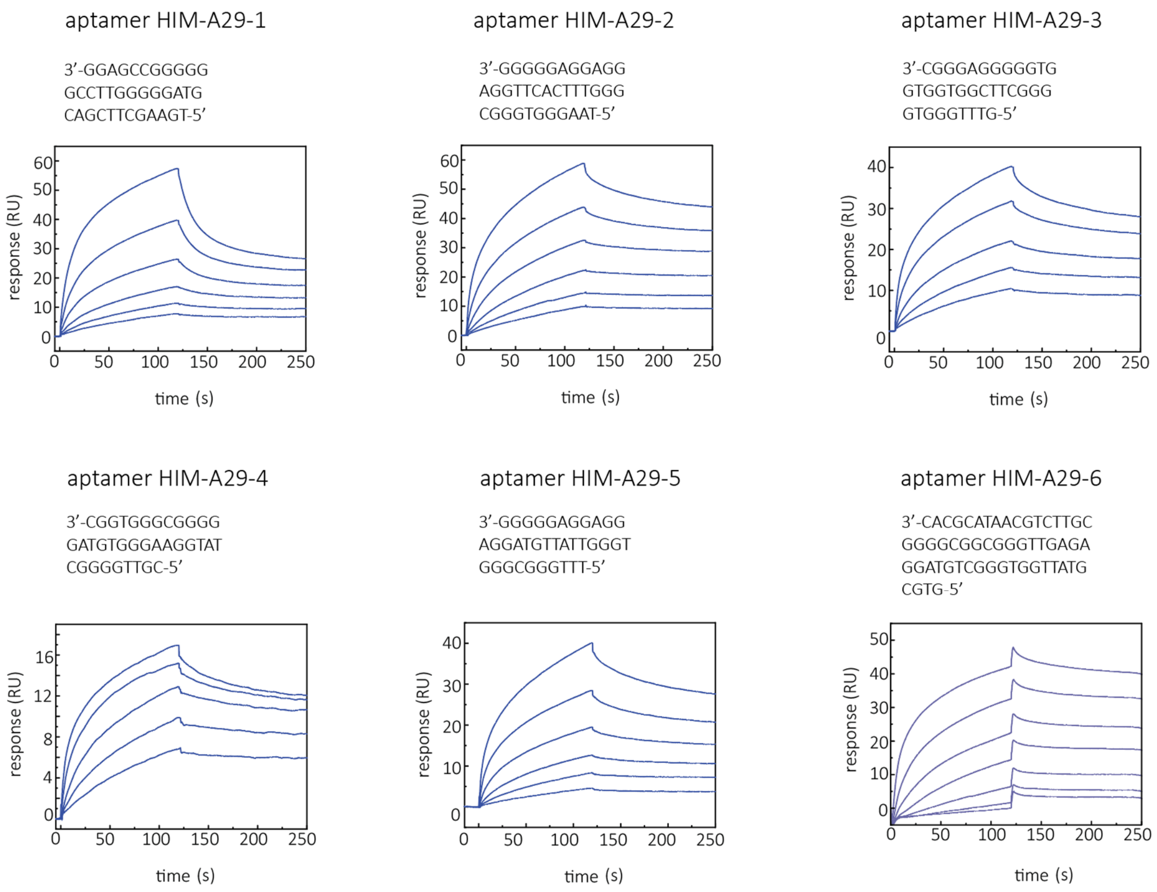

Fig. S2. SPR response curves of selected aptamers in the presence of different concentrations of A29 protein.

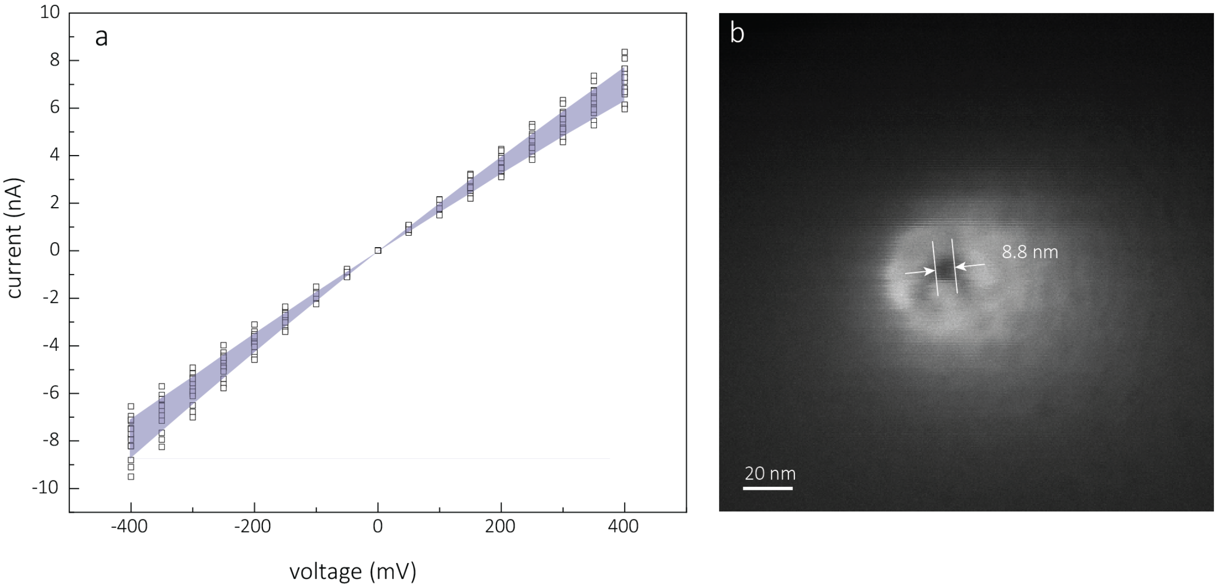

Fig. S3. Conductance and SEM characterization of the nanopipettes. (a) I-V curves of the nanopipettes were recorded before each experiment. By linear fitting, the I-V curves, the conductance of the nanopore was estimated to be G = 18.67 ± 1.82 nS (n = 20). The blue-shaded region indicates the standard deviation (n = 20). **(b)** SEM image showing the cross-section of a typical nanopipette (scale bar: 20 nm). The diameter was roughly 8.8 nm.

I-V characterization was performed to approximate the pore size and determine pore conductance prior to translocation experiments. The equation below was used to estimate the pore size.[5]

$$R_{\mathrm{p}} = \frac{1}{\kappa \pi r_{\mathrm{i}} \tan\theta} + R_{\mathrm{access}} \approx \frac{1}{\kappa \pi r_{\mathrm{i}} \tan\theta} + \frac{1}{4\kappa r_{\mathrm{i}}}$$

where R_{p} is the nanopipette resistance, r_{i} is the inner pipette radius, κ is the solution conductivity, and θ is the inner nanopipette half-cone angle. This equation estimated a pore diameter of 9 ± 2 nm, which was in good agreement with that obtained from SEM measurements.

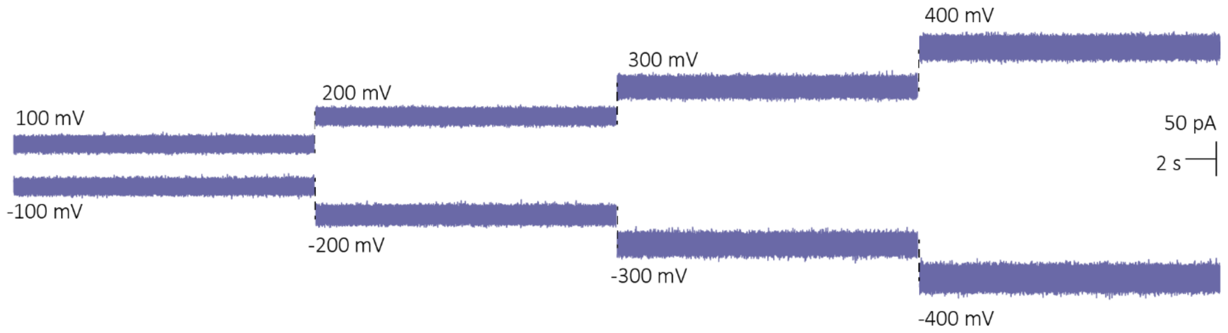

Fig. S4. Representative current-time traces for nanopore measurements in the presence of A29. Measurements were performed at an applied bias ranging from -400 mV to 400 mV. All the measurements were performed in 1 M LiCl and 1 M KCl electrolyte (5 mM $MgCl_2$, 10 mM Tris-HCl, 1 mM EDTA, pH = 8).

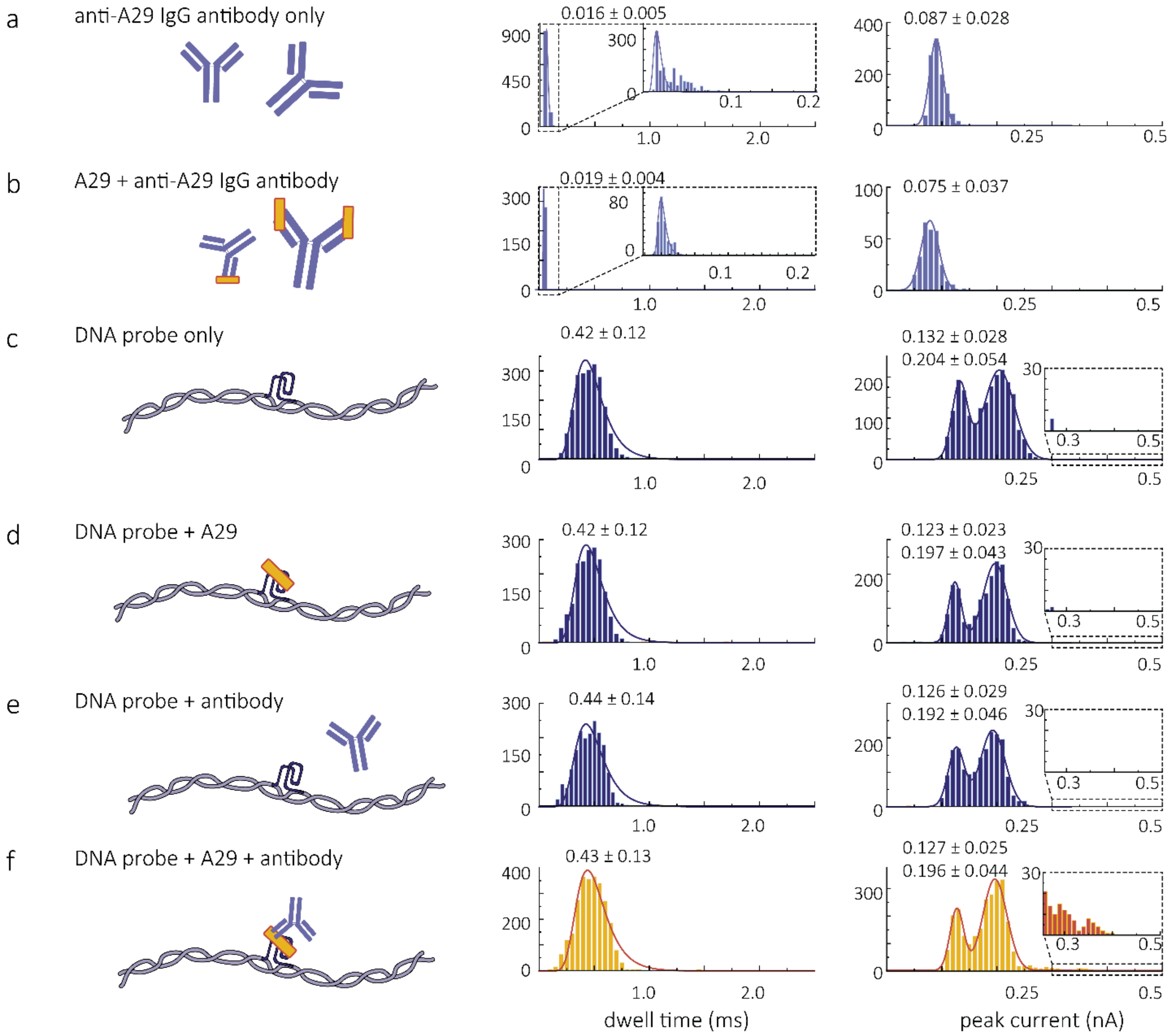

Fig. S5. Dwell time and peak height histograms for the translocation of (a) anti-A29 IgG antibody only, (b) A29 + anti-A29 IgG antibody, (c) DNA molecular probe only, (d) DNA molecular probe + A29 protein, (e) DNA molecular probe + antibody, and (f) DNA molecular probe + A29 + antibody.

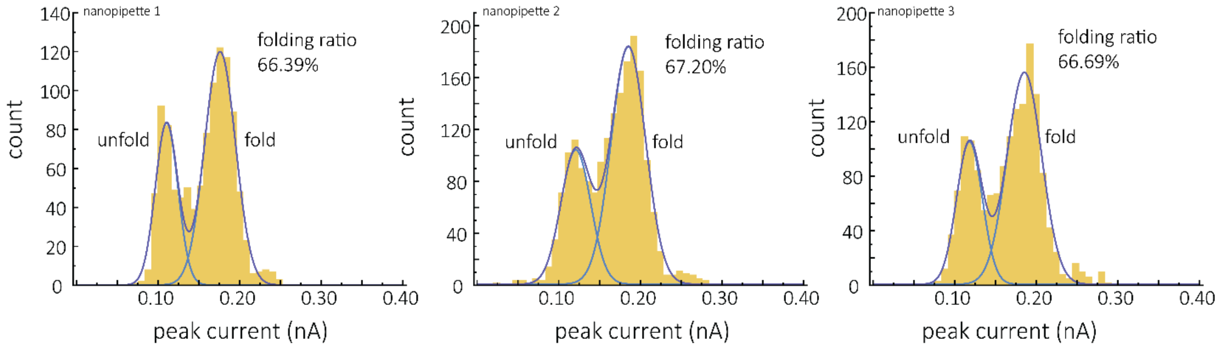

Fig. S6. Folding/unfolding ratio calculation based on 100 pM DNA molecular probe in 1M KCl and 1M LiCl.

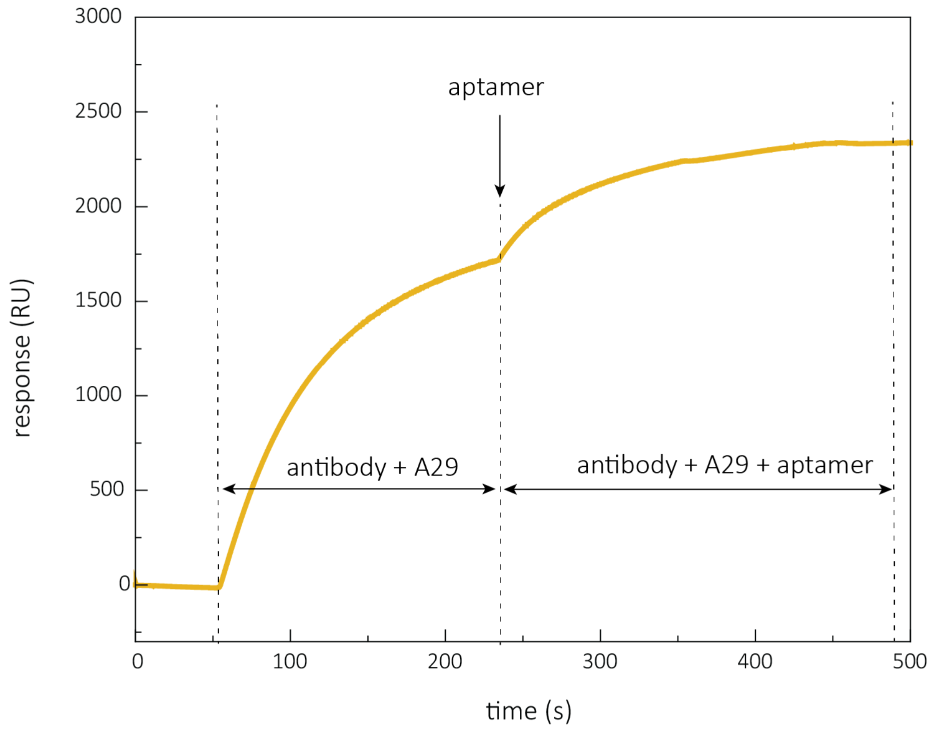

Fig. S7. SPR response curve for binding of A29 in the presence of antibody and aptamer (HIM-A29-6).

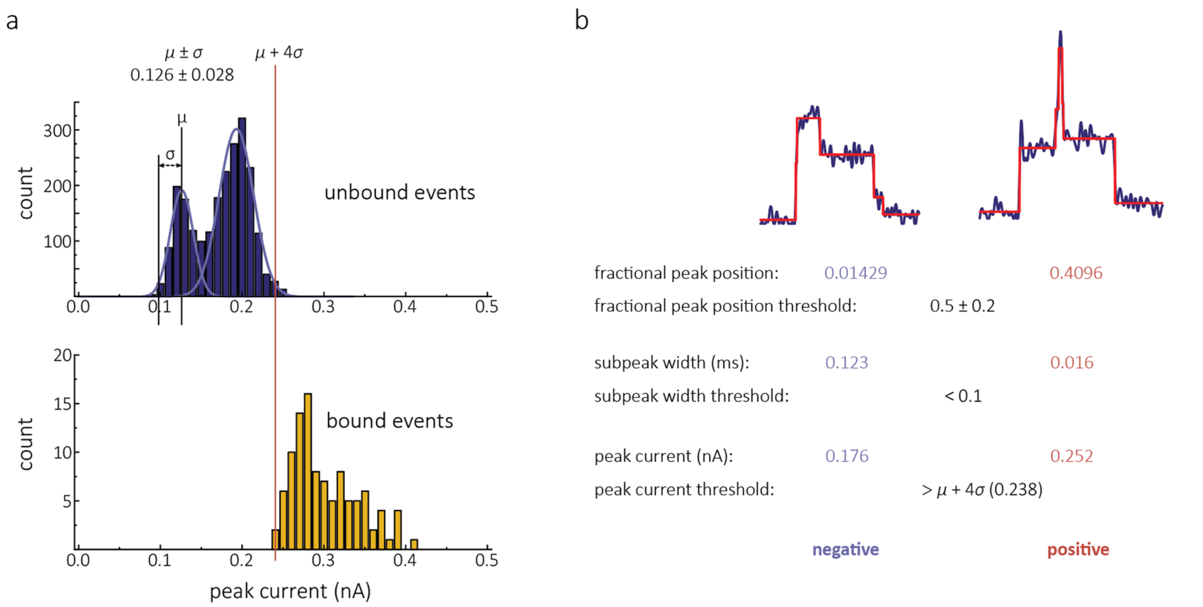

Fig. S8. Threshold for selection of subpeaks. (a) To set the threshold for subpeak selection, the mean value (μ) and standard deviation (σ) of the unfolded DNA level were calculated by fitting the distribution to a Gaussian distribution. A $\mu + 4\sigma$ threshold was used to classify positive subpeak events. (b) Comparison of a folded DNA event along with an event containing a positive subpeak.

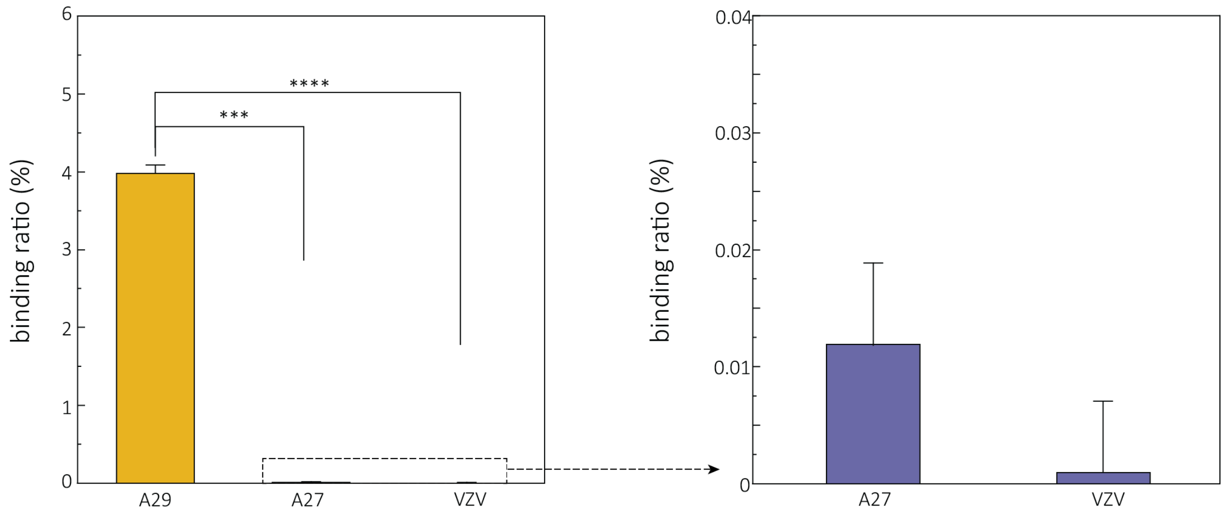

Fig. S9. Comparison of binding ratio for A27, VZV, and A29 protein in buffer. Statistical significance was tested using a two-tailed Student's t-test; ***P < 0.001, ****P < 0.0001.

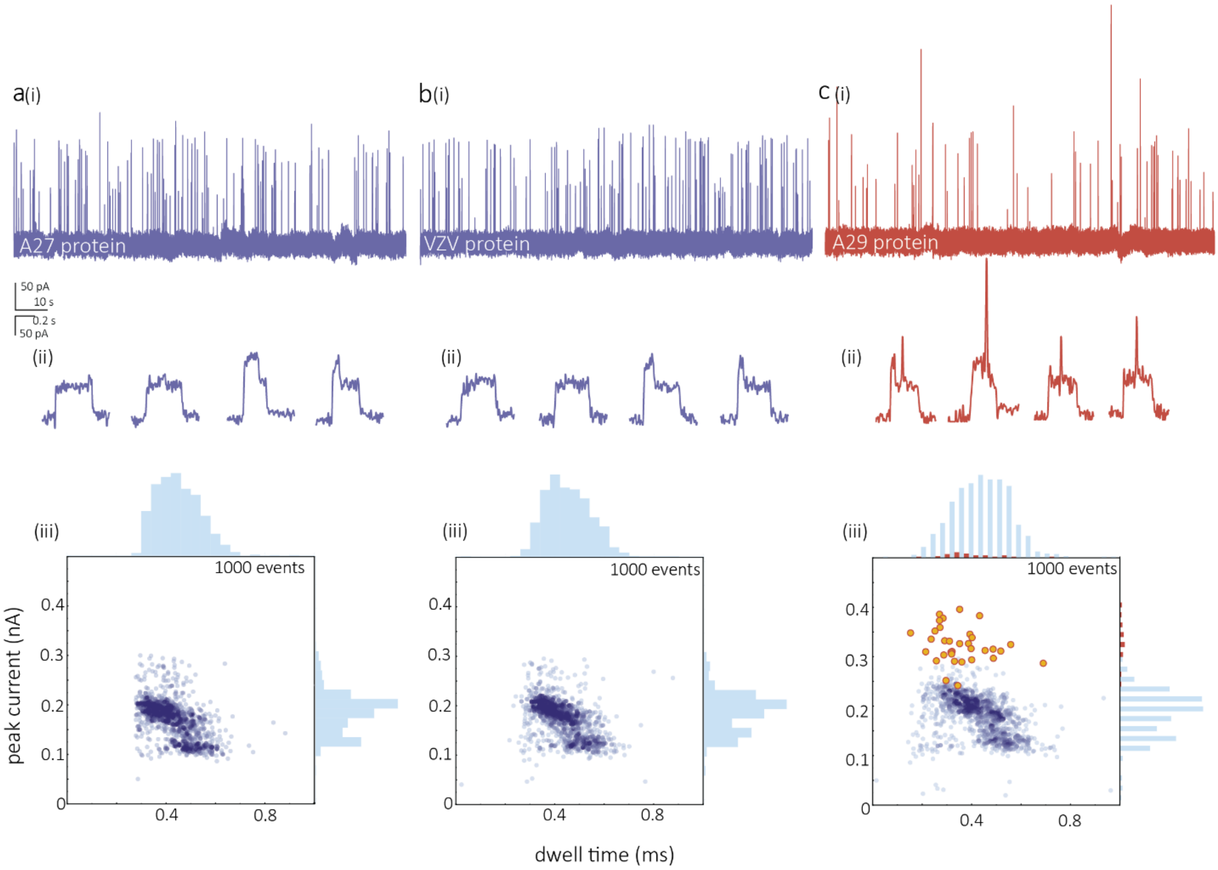

Fig. S10. Specificity in 5% human serum. (**a-c**) Representative current-time traces (i), typical events (ii), and statistics of dwell time and peak amplitude (iii) for the translocation of DNA molecular probes in the presence of vaccinia virus A27 protein (**a**), varicella zoster virus protein (**b**), and mpox A29 protein (**c**). The translocation events with positively classified subpeaks are highlighted in yellow (iii). The final concentrations of the DNA probe, target protein, and antibody were 100 pM, 1 nM, and 20 nM, respectively. All experiments were performed in 1 M LiCl and 1 M KCl electrolyte (5 mM $MgCl_2$, 10 mM Tris-HCl, 1 mM EDTA, pH = 8) mixed with 5% human serum under an applied bias of 300 mV.

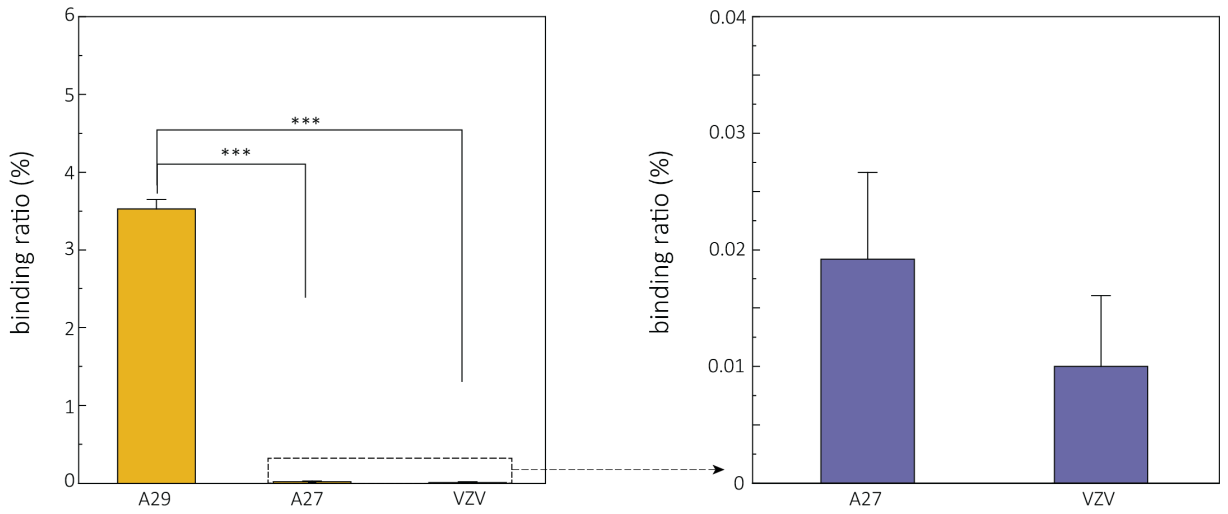

Fig. S11. Comparison of binding ratio for A27, VZV, and A29 protein in 5% human serum. Statistical significance was tested using a two-tailed Student's t-test; ***P < 0.001.

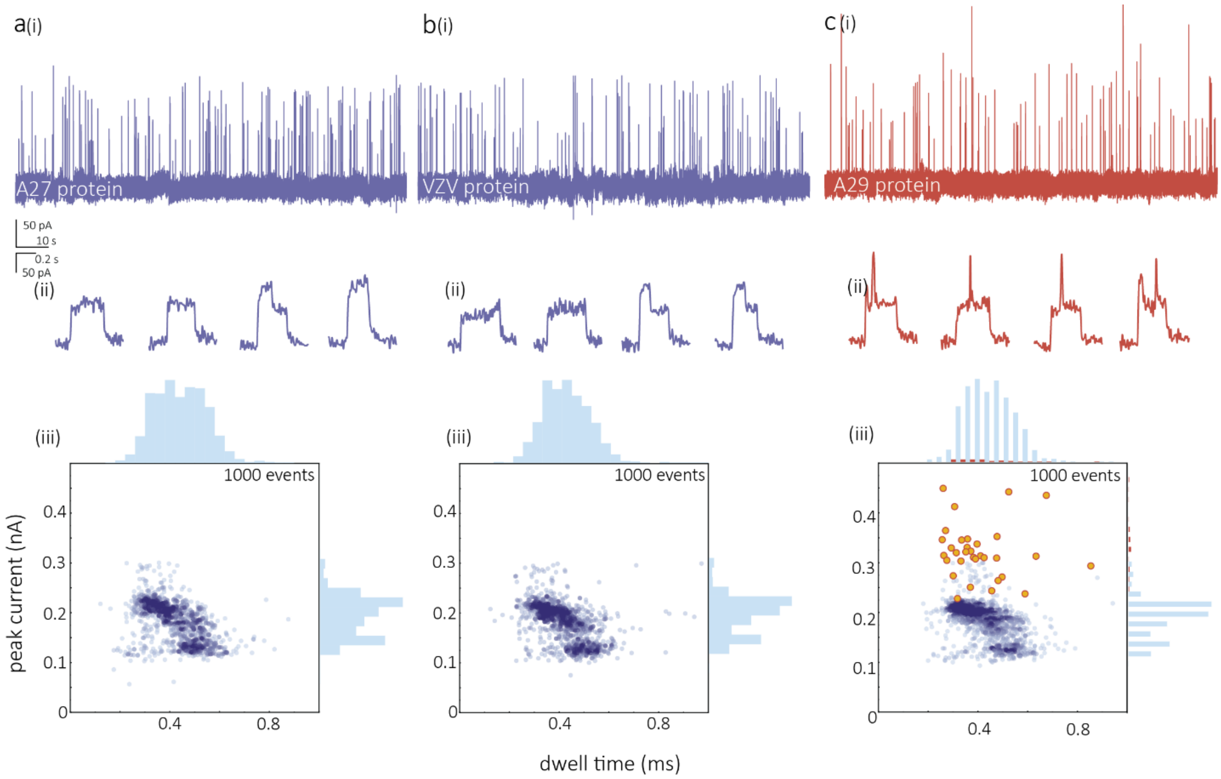

Fig. S12. Specificity in 5% human saliva. (**a-c**) Representative current-time traces (i), typical events (ii), and statistics of dwell time and peak amplitude (iii) for the translocation of DNA molecular probes in the presence of vaccinia virus A27 protein (**a**), varicella zoster virus protein (**b**), and mpox A29 protein (**c**). The translocation events with detected subpeak were highlighted in yellow (iii). The final concentrations of the DNA probe, target protein, and antibody were 100 pM, 1 nM, and 20 nM, respectively, in all experiments. All experiments were performed in 1 M LiCl and 1 M KCl electrolyte (5 mM $MgCl_2$, 10 mM Tris-HCl, 1 mM EDTA, pH = 8) mixed with 5% human saliva under an applied bias of 300 mV.

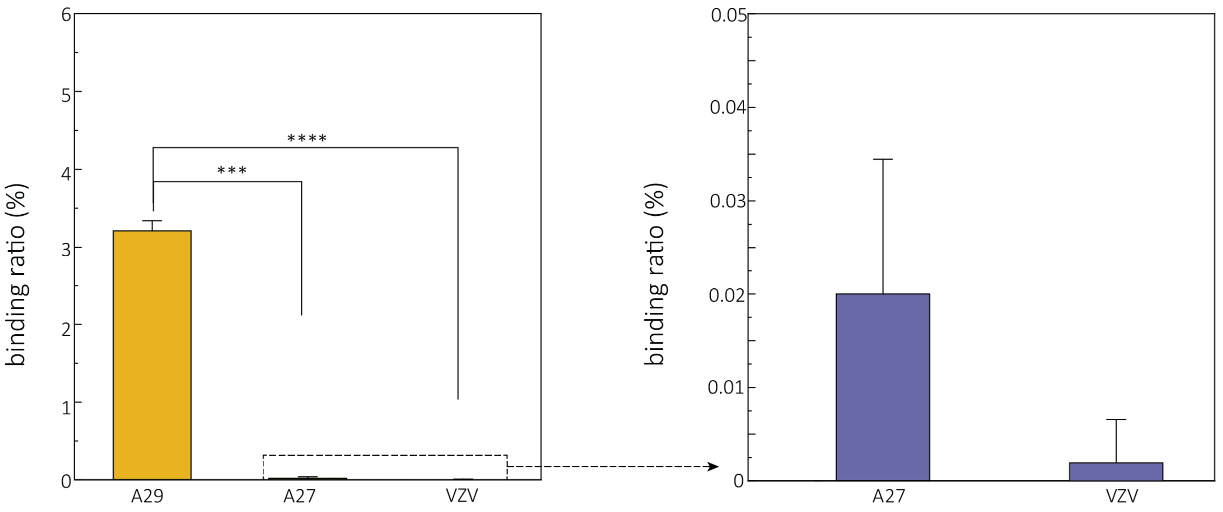

Fig. S13. Comparison of binding ratio for A27, VZV, and A29 protein in 5% human saliva. Statistical significance was tested using a two-tailed Student's t-test; ***P < 0.001, ****P < 0.0001.

Data Analysis

Single-molecule events were analyzed using the Nanopore App v7.17, a Matlab-based program by Prof. Joshua Edel. It offers the possibility to perform a comprehensive analysis of diverse types of single-molecule data traces. It facilitates tasks such as importing traces, resampling/refiltering, event identification, and exporting all relevant information.

Below is a summary of a typical workflow ranging from importing raw data to performing statistical analysis.

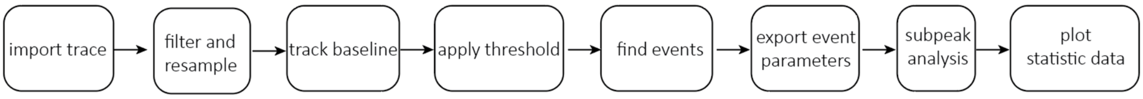

Step 1. Import raw trace

The Nanopore App supports several file formats, including ABF (Axon Digidata, Molecular Device), WCP (NI-DAQ, National Instruments), LOG, MAT (VC100, Chimera Instruments), DAT (eOne, Elements), and others. In the figure below, we have imported data related to detecting the A29 protein with the IgG antibody. This data was recorded using a Chimera VC100 amplifier at a sampling rate of 1 µs, with no filter applied.

Step 2. Resample and filter trace as needed

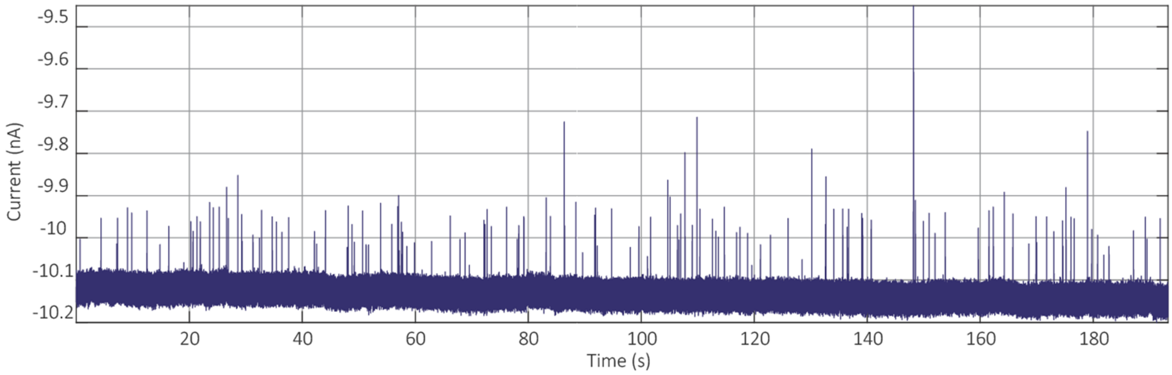

It is possible to perform resampling and refiltering to improve event discrimination. In this example, the trace was resampled at 1 µs and subjected to a low pass filter with a cutoff frequency of 50 kHz.

Step 3. Baseline tracking

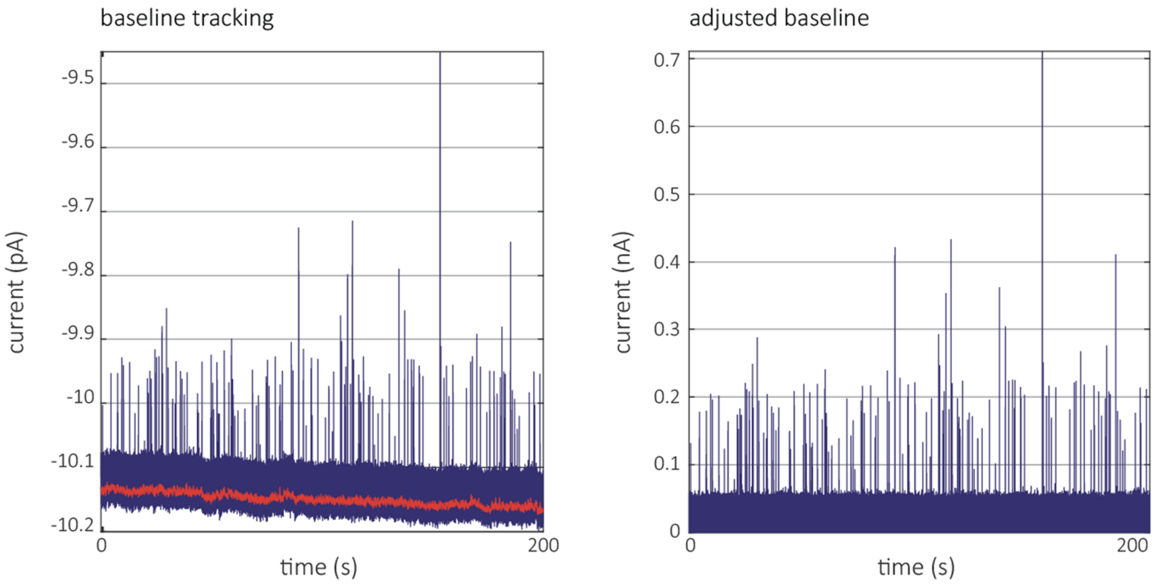

The baseline was tracked using a polynomial fit to compensate for signal fluctuations and improve event classification.

Step 4. Determination of the threshold for event classification

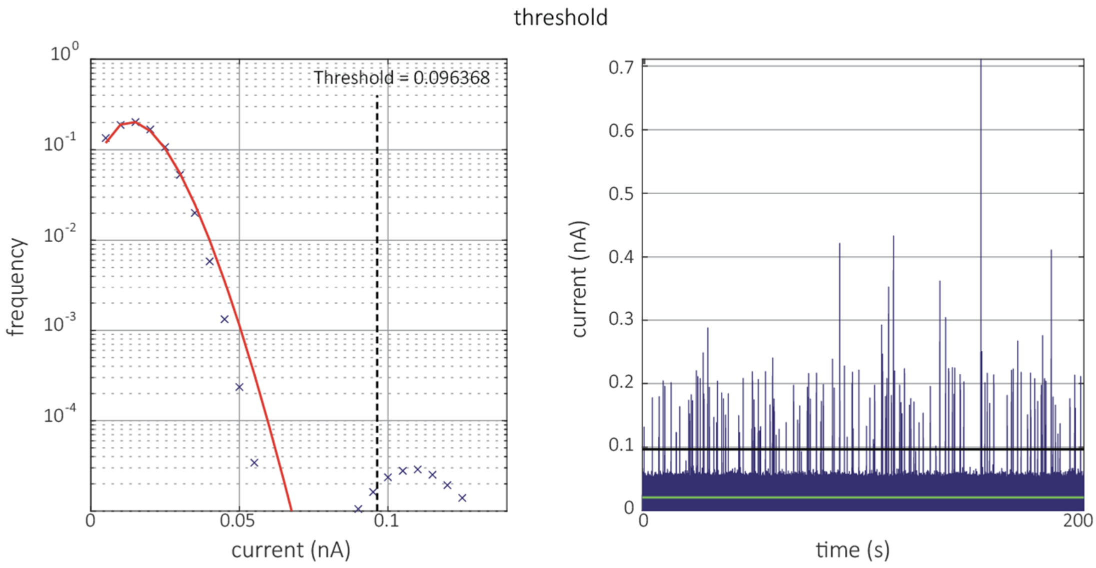

The signal was then histogrammed and fitted to a Poisson distribution. This could then be used to determine an upper threshold (represented by the black line) based on chosen number of standard deviations above the mean.

Step 5. Isolation of events

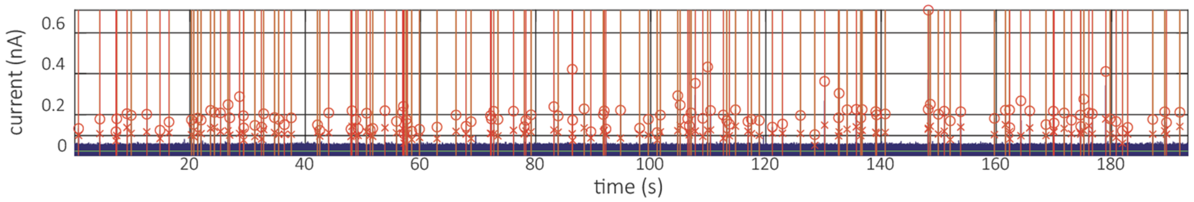

Based on the selected thresholds, events could be classified and isolated. Different thresholds, such as dwell time, peak current, and peak area, can also be applied as needed. This helps filter out noisy or irrelevant events, ensuring better event selection. A CUSUM fitting routine[6, 7] was used to fit each event. Information such as translocation time, peak amplitude, peak area, and subpeak information can be obtained as output parameters from the fit.

Step 6. Export event parameters

Subsequently, the peak amplitude, area, and width can be exported to the Matlab workspace for further analysis. The exported data can be plotted using various software tools such as Excel, Matlab, or OriginLab.

Step 7. Subpeak analysis

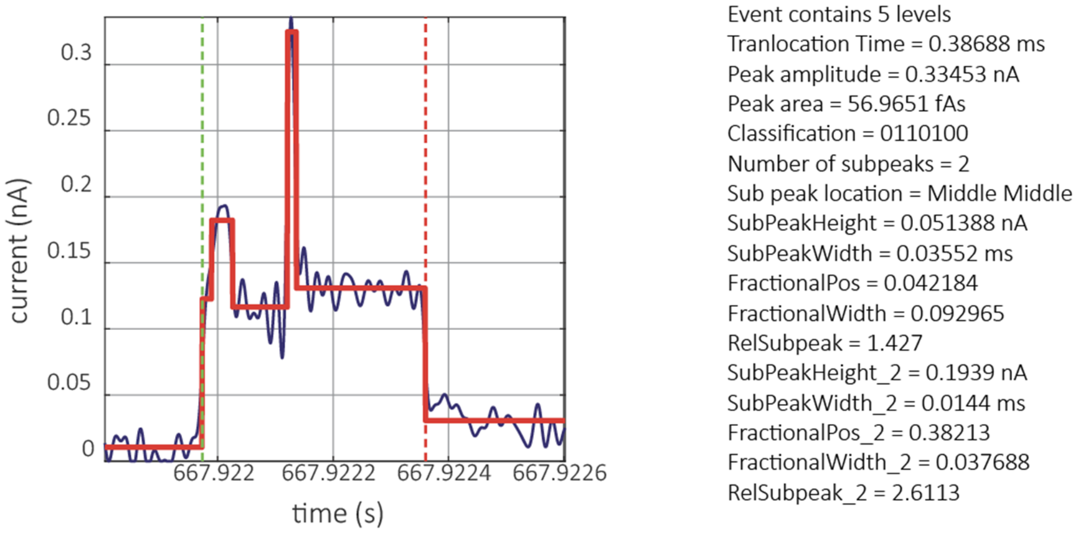

As an example, the output of information that could be obtained from an individual event is shown above. This information can also be exported to the Matlab workspace for further analysis.

Supplementary Tables

Table S1. Sequences of the initial library and primers used in SELEX.

DNA species	Sequences (5’ to 3’)
DNA library	TCCAGCACTCCACGCATAACNNNNNNNNNNNNNNNNNNNNNNNNNNNNNNNN NNNNGTTATGCGTGCTACCGTGAA
Primer-forward	TCCAGCACTCCACGCATAAC
Primer-reverse	TTCACGGTAGCACGCATAAC
Primer-polyA	AAAAAAAAAAAAAAAAAAA-spacer18-TTCACGGTAGCACGCATAAC

Table S2. Sequences of the selected aptamer with strong affinity.

Aptamer	Sequences (5’ to 3’)	K_d (nM)
HIM-A29-1	AGCACTCCACGCATAACGGGGTCTGGTGGTCTGGCTGGTTTGGGGGTTTGGTCGT TATGCGTGCTACCGT	10.8
HIM-A29-2	AGCACTCCACGCATAACGGAGCCGGGGGGCCTTGGGGGATGCAGCTTCGAAGTG TTATGCGTGCTACCGT	35.4
HIM-A29-3	AGCACTCCACGCATAACGGGGGAGGAGGAGGTTCACTTTGGGCGGGTGGGAATG TTATGCGTGCTACCGT	12.4
HIM-A29-4	AGCACTCCACGCATAACGGGGGAGGAGGAGGATGTTATTGGGTGGGCGGGTTTG TTATGCGTGCTACCGT	18.6
HIM-A29-5	AGCACTCCACGCATAACCGGTGGGCGGGGGATGTGGGAAGGTATCGGGGTTGCG TTATGCGTGCTACCGT	8.49
HIM-A29-6	CACGCATAACGTCTTGCGGGGCGGCGGGTTGAGAGGATGTCGGGTGGTTATGCG TG	5.63

Table S3. Aptamer probes and the complementary sequences in DNA molecular probe.

Oligos	Sequences (5' to 3')	Specification
Aptamer-1-probe	5'-Phos- CAC TGC GCC GGT ATC GAT ATA TTT TTT CAC GCA TAA CGT CTT GCG GGG CGG CGG GTT GAG AGG ATG TCG GGT GGT TAT GCG TG - 3'	Complementary sequences to DNA carrier are in green. Aptamer sequences are in red.
Aptamer-2-probe	5'-Phos- CAC TGC GCC GGT ATC GAT ATA TTT TTT AGC ACT CCA CGC ATA ACC GGT GGG CGG GGG ATG TGG GAA GGT ATC GGG GTT GCG TTA TGC GTG CTA CCG T- 3'	Complementary sequences to DNA carrier are in green. Aptamer sequences are in red.
48-base bleached sequences in 9.1 kbp	5'- AAA CCA CTC AAG TTT GCC AAC CAA ATG **TAT ATC GAT ACC GGC GCA GTG** - 3'	Bases in bold are sequences complementary to the designed probe.

References

(1) Cai, S.; Pataillot-Meakin, T.; Shibakawa, A.; Ren, R.; Bevan, C. L.; Ladame, S.; Ivanov, A. P.; Edel, J. B. Single-molecule amplification-free multiplexed detection of circulating microRNA cancer biomarkers from serum. *Nat. Commun.* **2021**, *12*, 3515.
(2) Cai, S.; Sze, J. Y. Y.; Ivanov, A. P.; Edel, J. B. Small molecule electro-optical binding assay using nanopores. *Nat. Commun.* **2019**, *10*, 1797.
(3) Wang, X.; Wilkinson, M. D.; Lin, X.; Ren, R.; Willison, K. R.; Ivanov, A. P.; Baum, J.; Edel, J. B. Single-molecule nanopore sensing of actin dynamics and drug binding. *Chem. Sci.* **2020**, *11*, 970-979.
(4) Sze, J. Y. Y.; Ivanov, A. P.; Cass, A. E. G.; Edel, J. B. Single molecule multiplexed nanopore protein screening in human serum using aptamer modified DNA carriers. *Nat. Commun.* **2017**, *8*, 1552.
(5) Perry, D.; Momotenko, D.; Lazenby, R. A.; Kang, M.; Unwin, P. R. Characterization of Nanopipettes. *Anal. Chem.* **2016**, *88*, 5523-5530.
(6) Forstater, J. H.; Briggs, K.; Robertson, J. W. F.; Ettedgui, J.; Marie-Rose, O.; Vaz, C.; Kasianowicz, J. J.; Tabard-Cossa, V.; Balijepalli, A. MOSAIC: A Modular Single-Molecule Analysis Interface for Decoding Multistate Nanopore Data. *Anal. Chem.* **2016**, *88*, 11900-11907.
(7) Raillon, C.; Granjon, P.; Graf, M.; Steinbock, L. J.; Radenovic, A. Fast and automatic processing of multi-level events in nanopore translocation experiments. *Nanoscale* **2012**, *4*, 4916-4924.
